# Feeding and reproductive parameters of adult female *Ixodes scapularis* (Acari: Ixodidae) and *Amblyomma americanum* parasitizing white-tailed deer *(Odocoileus virginianus*)

**DOI:** 10.1093/jme/tjad144

**Published:** 2023-10-28

**Authors:** Alec S Baker, Kelly A Persinger, Pia U Olafson, Albert O Mulenga, Tammi L Johnson

**Affiliations:** Department of Rangeland, Wildlife and Fisheries Management, Texas A&M University, 495 Horticulture Road, College Station, TX 77843, USA; Texas A&M AgriLife Research, 1619 Garner Field Road, Uvalde, TX 78801, USA; Texas A&M AgriLife Research, 1619 Garner Field Road, Uvalde, TX 78801, USA; Livestock Arthropod Pests Research Unit, USDA-ARS, 2700 Fredericksburg Road, Kerrville, TX 78028, USA; Department of Veterinary Pathobiology, School of Veterinary Medicine and Biomedical Sciences, Texas A&M University, Veterinary Medical Sciences Building, 400 Raymond Stotzer Pkwy #4467, College Station, TX 77843, USA; Department of Rangeland, Wildlife and Fisheries Management, Texas A&M University, 495 Horticulture Road, College Station, TX 77843, USA; Texas A&M AgriLife Research, 1619 Garner Field Road, Uvalde, TX 78801, USA

**Keywords:** blacklegged tick, experimental infestation, lone star tick, white-tailed deer

## Abstract

White-tailed deer *Odocoileus virginianus* (Zimmermann) (Artiodactyla: Cervidae) are the main host for adult *Ixodes scapularis* Say (Acari: Ixodidae) (blacklegged tick) and all stages of *Amblyomma americanum* Linnaeus (Acari: Ixodidae) (lone star tick). However, literature describing the feeding and reproductive parameters of these tick species when feeding on this host is limited. We experimentally infested white-tailed deer with adult pairs of either *I. scapularis* or *A. americanum* to improve our understanding of these tick–host relationships. Our study used tick-naïve white-tailed deer and restricted host grooming throughout the infestation. For *I. scapularis*, the days to repletion (mean ± SE, 6.04 ± 0.07), engorgement weight of replete females (0.20 ± 0.0032 g), duration of oviposition (32 ± 0.45 d), egg mass weight (0.10 ± 0.0027 g), and number of eggs laid per tick (1,803.00 ± 49.00) were recorded. Data from *A. americanum* were also recorded, including days to repletion (11.00 ± 0.063), engorgement weight of replete females (0.63 ± 0.025 g), duration of oviposition (37.00 ± 1.30 d), egg mass weight (0.34 ± 0.017 g), and number of eggs laid per tick (5,873.00 ± 291.00). These biological parameter data could be used as variables in models (e.g., LYMESIM 2.0) to determine how white-tailed deer influence *I. scapularis* and *A. americanum* populations in nature, and to evaluate the protective efficacy of tick-antigen-based antitick vaccines.

## Introduction

In the United States, white-tailed deer (WTD) *Odocoileus virginianus* (Zimmerman) (Artiodactyla: Cervidae) are the host of various ectoparasites, including ticks, mites, and deer keds (Poh et al. 2022). WTDs are the main host for adult *Ixodes scapularis* Say (Acari: Ixodidae) (blacklegged tick), all life stages of *Amblyomma americanum* Linnaeus (Acari: Ixodidae) (lone star tick), as well as other ticks of medical and veterinary importance (Rand et al. 2000, Stafford III and Williams 2017, Tsao et al. 2021). In the 20th century, the recovery and continued population expansion of WTD from near extirpation in the United States allowed ticks to increase in abundance and expand geographically (Tsao et al. 2021). These increases are correlated with the rise of various tick-borne diseases, the pathogens of which are known to be transmitted by *I. scapularis*, *A. americanum*, and other species of ticks (Childs and Paddock 2003, Paddock and Yabsley 2007). While WTD do not support the enzootic cycles of many known tick-borne pathogens, including *Borrelia burgdorferi* sensu stricto, the causative agent of Lyme disease (Tsao et al. 2021), they are the main reservoir host of *Ehrlichia chaffeensis* and *Ehrlichia ewingii*, the pathogens that cause human monocytic ehrlichiosis (Paddock and Yabsley 2007). Furthermore, WTDs have aided in the spread and establishment of several invasive tick species, including cattle fever ticks [*Rhicephalus* (*Boophilus*) *annulatus* Say and *Rhicephalus (Boophilus) microplus* Canestrini], Gulf Coast ticks (*Amblyomma maculatum* Koch), and the recently introduced Asian longhorned tick (*Haemaphysalis longicornis* Neumann) (Tsao et al. 2021).

Eleven of the 16 most prevalent human tick-borne disease agents in the United States are transmitted by *I. scapularis* and *A. americanum* (https://www.cdc.gov/ticks/diseases/index.html) and reducing the potential for pathogen transmission by these tick species may involve targeting both their off-host and on-host stages. Given the importance of WTD to their lifecycle, these cervid hosts are frequently the target of efforts to control on-host populations of *I. scapularis* and *A. americanum*. Such efforts include the delivery of topical and systemic acaricides, physical barriers, and WTD population reduction (Stafford III and Williams 2017). Improving and supplementing existing tick control strategies and developing additional approaches would benefit from a thorough understanding of the biological parameters of *I. scapularis* and *A. americanum* parasitizing WTD; however, there are limited reports of feeding and reproductive potential of adults of these tick species on a WTD host (Koch 1988, Hornbostel et al. 2004). The objective of this research was to define baseline feeding and reproductive parameters of adult *I. scapularis* and *A. americanum* using tick-naïve captive WTD as a host. The results from this research will fill a gap in the scientific literature and better inform future methodologies targeting WTD for controlling *I. scapularis* and *A. americanum*.

## Materials and Methods

### White-tailed Deer Hosts

Female WTD fawns (*N* = 8) ranging from 2 to 6 days old were obtained in 2020, and similarly aged female WTD fawns (*N* = 3) were obtained in 2021 from a private breeder (Guadalupe County, TX). The fawns were bottle-raised for the first 4 months of life at the Texas A&M AgriLife Research and Extension Center (Uvalde, TX). During this period, the fawns were habituated to people and acclimated to a lift chute (Pound et al. 2003) designed for restraining and handling WTD. This approach reduced stress on the deer while being handled for this research. All animal usage was approved by the Texas A&M Agricultural Animal Care and Use Committee protocols #2020-018A and #2020-028A.

### Experimental Infestation of WTD with *I. scapularis* and *A. americanum*

During February 2021, we infested the first cohort of fawns (*N* = 8; approx. 8 months old) with premated, adult *I. scapularis* (*N* = 100; 50 mated pairs per WTD), obtained from the Oklahoma State University Tick Rearing Laboratory. This colony has been maintained since 1991, originated from *I. scapularis* collected in Oklahoma, and is supplemented with engorged females collected from naturally infested university-owned cattle yearly. To premate the ticks, 50 male and 50 female ticks were combined 24–48 h before the infestation started in a single 50-mL conical centrifuge tube with 10 air holes created in the screw-capped lid (punctured the lid using an 18G hypodermic needle). During May 2022, we infested the second cohort of fawns (*N* = 3; approx. 10 months old) with adult *A. americanum* (*N* = 100; 50 males and 50 females per WTD) obtained from the Knipling-Bushland US Livestock Insect Research Laboratory (USDA-ARS, Kerrville TX). This colony has been maintained since 2006 and originated from the Kerr Wildlife Management Area near Hunt, TX.

The infestation followed the protocol described in Baker et al. (2023). Briefly, the infestation was restricted to the area at the base of the neck and between the shoulders to reduce the probability of the ticks being removed by host grooming. One day prior to tick infestations, this area was shaved and a ~100-mm × ~300-mm cotton stockinette (“tick patch”) was adhered to the area using liquid bonding cement (TORBOT Group, Inc.). After ticks were applied to the patched area, the tick-infested WTD were housed in individual pens throughout the infestation to prevent interhost grooming. Beginning 5 days post-infestation, infested WTD were checked daily for the presence of replete female ticks. Replete female ticks were removed from the stockinette daily until all ticks had detached or died. Female ticks were occasionally found destroyed inside the patch, and these dead ticks were removed and discarded. These were not included in the calculations, as their deaths resulted from external events. There were instances of female ticks that engorged but died before dropping from the host; these females were physically removed. Any remaining male ticks that were either questing or attached were physically removed from the patch after all females had dropped/were removed. These males were discarded in ethanol. After all ticks were removed from the tick patch, the patch was removed using TORBOT Solvent Adhesive Remover.

### Tick Biological Parameters

Definitions for all biological parameters evaluated are presented in Table 1. The number of days from initial infestation to repletion was recorded, and the percentage of ticks that fed to repletion was calculated as ((number of replete females/50) × 100). Each detached female tick was transferred to a preweighed 4-dram glass shell vial to determine the weight of each replete tick (grams) and the drams were incubated in a humidity-controlled chamber to await oviposition (CARON Insect Rearing Chamber; 90% relative humidity (RH), 22°C ± 1°C, and 16:8 [photoperiod; light:dark hours]). Ticks were monitored daily to determine the time to onset of oviposition (preoviposition period) and duration of oviposition (days). When egg laying ceased, the spent female was removed, and the dram was weighed to determine the egg mass weight (grams). Female tick mortality during oviposition was recorded; however, only ticks that died before the onset of oviposition were included when calculating percent mortality. Bloodmeal conversion to egg mass was calculated using the formula:

**Table 1.** Biological parameters estimated for female *Ixodes scapularis* and *Amblyomma americanum* fed on tick-naïve white-tailed deer restricted from grooming

Parameter	Definition/Quantification
Days to repletion	Total days from when tick was placed on host until detached from host
Percent repletion (%)	Number of ticks that fed and detached from host
Percent mortality (%)	Percent of female ticks that died prior to oviposition per host
Engorged female weight (g)	Weight of female tick immediately post-detachment
Preoviposition period (days)	The period between adult female engorgement and egg laying
Duration of oviposition (days)	Total days from time of the first eggs laid until no additional eggs detected on female
Pre-eclosion period (days)	The period between end of egg laying and larval hatching
Egg mass weight (g)	Weight of egg mass
Bloodmeal conversion to egg mass (%)	Weight of egg mass divided by the weight of engorged female * 100
Eggs laid per tick	Average number of larvae plus unhatched eggs per cell counted in 25 randomly chosen cells when the entire contents of the egg mass was distributed on a 100-cell grid * 100
Hatch success (%)	Number of egg masses that hatched larvae divided by total number of egg masses laid *100
Larvae hatched per tick	Average number of larvae per cell counted in 25 randomly chosen cells when the entire contents of the egg mass was distributed on a 100-cell grid * 100
Percent hatch (%)	Number of larvae hatched divided by the total eggs laid * 100


$$\begin{array}{lll} & Bloodmeal\;conversion\;to\;egg\;mass\; & =\left(\frac{weight\;of\;egg\;mass\;\left(g\right)}{weight\;of\;engorged\;female\left(g\right)}\right)*100. \end{array}$$

The completion of oviposition was designated when the female was determined to be dead (spent). Sixty days after completion of oviposition, 70% ethanol was added to each dram to preserve all hatched larvae and remains of the egg mass; the hatched larvae and unhatched eggs were enumerated as follows: The dram was shaken vigorously, the contents dispensed onto a 10 × 10 cell grid, a subset of cells (*n* = 25) were randomly selected and viewed under a microscope, and the total number of larvae and unhatched eggs per cell was recorded. The average of the 25 cells * 100 was used to estimate the number of eggs laid and the number of larvae hatched, and these were used to calculate “percent larval hatch” using the formula:


$$\begin{array}{lll} & Percent\;larval\;hatch\;of\;egg\;mass\; & =\left(\frac{number\;of\;larvae\;hatched}{number\;of\;larvae\;hatched\;+\;number\;of\;unhatched\;eggs}\right)*100. \end{array}$$

Data were evaluated using GraphPad Prism 9.3.1 (GraphPad Software, LLC, San Diego, CA).

## Results

Replete *I. scapularis* females were recovered over the course of 5–11 days post-infestation (mean ± SE, 6.04 ± 0.07). On average, greater than 80% of female ticks (*N* = 327/400) fed and naturally detached from each host, ranging from 58.00% to 98.00% per WTD (81.50 ± 4.36). Of the remaining 327 ticks, mortality of *I. scapularis* females during the preoviposition period ranged from 0.00% to 41.03% per WTD (18.70 ± 4.57). The engorgement weight of female ticks that fed to repletion (*n* = 327) ranged from 0.02 to 0.43 g (0.20 ± 0.0032), and of all *I. scapularis* combined that fed to repletion 83.49% survived to oviposition (*n* = 273/327). The preoviposition period ranged from 8 to 31 d post-repletion (11.07 ± 0.16), and the duration of oviposition ranged from 14 to 46 days (33.49 ± 0.44). Egg masses ranged in weight from 0.0042 to 0.18 g (0.098 ± 0.002), and the calculated bloodmeal conversion to egg mass ranged from 3.30% to 65.00% (45.82 ± 0.76). Larval hatch success across all egg masses, as defined in Table 1, ranged from 88.00% to 100.00% (96.23 ± 1.46). The estimated number of eggs laid per tick ranged from 4 to 4,036 (1,803 ± 48.81) and the number of larvae hatched per egg mass ranged from 0 to 3,684 (1,367 ± 52.67) with an estimated percent larval hatch ranging from 0.00% to 100.00% (68.77 ± 1.84). These results are summarized in Table 2.

**Table 2.** Feeding and reproductive parameters of adult female *Ixodes scapularis* on tick-naïve white-tailed deer restricted from grooming

Parameter	_ $\bar{x}$ _	Standard error	95% CI	Sample size
Days to repletion	6.04	±0.07	5.92–6.17	327
Percent repletion (%)	81.50	±4.36	71.20–91.80	8
Percent mortality (%)	18.70	±4.57	7.89–29.52	8
Engorged female weight (g)	0.20	±0.0032	0.19–0.21	327
Preoviposition period (d)	11.07	±0.16	10.75–11.39	273
Duration of oviposition (d)	33.49	±0.44	32.63–34.36	268
Pre-eclosion period (d)	49.98	±0.29	49.41–50.55	251
Egg mass weight (g)	0.098	±0.0023	0.094–0.103	269
Bloodmeal conversion to egg mass (%)	45.82	±0.76	44.32–47.32	269
Eggs laid per tick	1,803	±48.91	1,707–1,900	268
Hatch success (%)	96.23	±1.46	91.73–97.46	8
Larvae hatched per tick	1,367	±52.67	1,269–1,477	265
Percent hatch (%)	68.77	±1.84	64.77–72.02	265

The length of time for adult female *A. americanum* to feed to repletion ranged from 8 to 12 d post-infestation (mean ± SE, 10.50 ± 0.06). Greater than 90% of adult females (*N* = 139/150) fed and naturally detached, and the percentage of female ticks that fed to repletion per WTD ranged from 88.00% to 96.00% (92.67 ± 2.40). Percent mortality of adult female *A. americanum* prior to oviposition per WTD ranged from 10.00 to 76.03 (34.00 ± 21.07). The engorgement weight of fed female ticks (*n* = 139) ranged from 0.0078 to 1.20 g (0.63 ± 0.03), and of all *A. americanum* combined that fed to repletion 70.50% survived to oviposition (*n* = 98/139). The preoviposition period ranged from 12 to 60 d post-repletion (15.72 ± 0.58), and the duration of oviposition ranged from 1 to 52 d (37.28 ± 1.30). Egg masses ranged in weight from 0.0047 to 0.86 g (0.34 ± 0.02), and the estimated number of eggs laid per tick ranged from 28.00 to 11,688.00 (5,873 ± 291.20) with a 0.49%–90.00% range in bloodmeal conversion to egg mass (47.65 ± 1.51). Larval hatch success across all egg masses, as defined in Table 1, ranged from 87.50% to 100.00% (95.08 ± 3.84). The estimated percent larval hatch per egg mass ranged from 0.00% to 99.00% (66.68 ± 3.20), and the number of larvae hatched per egg mass ranged from 0.00 to 10,576.00 (4,029 ± 286.90). These results are summarized in Table 3.

**Table 3.** Feeding and reproductive parameters of *Amblyomma americanum* on tick-naïve white-tailed deer restricted from grooming

Parameter	_ $\bar{x}$ _	Standard error	95% CI	Sample size
Days to repletion	10.50	±0.06	10.38–10.63	139
Percent repletion (%)	92.67	±2.40	–^a^	3
Percent mortality (%)	34.00	±21.07	–^a^	3
Engorged female weight (g)	0.63	±0.03	0.59–0.68	139
Preoviposition period (d)	15.72	±0.58	14.57–16.88	98
Duration of oviposition (d)	37.28	±1.30	34.70–39.85	98
Pre-eclosion period (d)	15.26	±1.26	12.75–17.78	84
Egg mass weight(g)	0.34	±0.02	0.31–0.38	98
Bloodmeal conversion to egg mass (%)	47.65	±1.51	44.66–50.65	98
Eggs laid per tick	5,873	±291.20	5,295–6,451	96
Hatch success (%)	95.08	±3.84	–^a^	3
Larvae hatched per tick	4029	±286.90	3,459–4,599	96
Percent hatch (%)	66.68	±3.20	60.00–73.00	96

^a^Due to small sample sizes, the 95% CI was not calculated.

## Discussion

Biological parameters of *I. scapularis* and *A. americanum* development that are used in simulation models have relied on data of engorged females collected from a variety of hosts, including cattle, dogs, raccoons, and white rabbits (Haile and Mount 1987, Mount et al. 1997, Ogden et al. 2005, Ludwig et al. 2015, and references therein). WTDs have been targeted for the control of on-host tick populations; however, the literature regarding the feeding and reproductive parameters of *I. scapularis* and *A. americanum* when parasitizing this host are limited. Hornbostel et al. (2004) collected *I. scapularis* from hunter-harvested WTD and reported data on female engorgement weight, days to onset of oviposition, duration of oviposition, and egg mass weight. While the mean engorgement weight of adult female *I. scapularis* was comparable to that in our study (0.20 g), several other biological parameters were in contrast. For example, the mean days to onset and duration of oviposition for *I. scapularis* from hunter-harvested hosts were approximately 4 times (mean ± SE, 41.00 ± 9.00 versus 11.07 ± 0.16 d) and nearly half (17.00 ± 2.00 versus 33.49 ± 0.44 d) as long as in our study, respectively. Furthermore, the mean egg mass weight was 5 times less from *I. scapularis* of hunter-harvested hosts than in our confined study (0.0283 ± 0.0008 versus 0.098 ± 0.0023 g), and the field-collected ticks were less efficient at converting bloodmeal to an egg mass (15.00 ± 4.10% versus 45.82 ± 0.76%). These differences could be attributed to several factors: the inclusion of nearly replete ticks from hunter-harvested WTD (removal too early), variation in the temperature at which engorged females were incubated, and artifacts of acquired host immunological resistance. Hornbostel et al. (2004) incubated engorged females at 26°C versus 22°C (this study), and slight differences in temperature are known to directly impact the preoviposition and oviposition periods (Ogden et al. 2004). Acquired host resistance to ticks develops over the course of repeated exposure to blood-feeding ectoparasites. This phenomenon has been documented at the tick–host interface for several mammalian hosts (Wikel 1996), and it can manifest as reductions in egg mass weights and blood conversion efficiency. In addition, our results are based on experimentally infested naïve captive WTD from Texas with *I. scapularis* from a colony originating from field collections in the southeastern United States. Variation between our studies, then, may also be related to different clades analyzed in Hornbostel et al. (2004) and the current study (i.e., southern WTD, southern clade of *I. scapularis* versus northern WTD, and northern clade of *I. scapularis*). For this reason, we interpret comparisons of feeding and reproductive parameters between studies with the understanding that they may differ depending on combinations of host–tick geographic origins/clade.

Koch (1988) described parameters for all life stages of *A. americanum* obtained from WTD that were experimentally infested, and the host was allowed to naturally groom itself. Several biological parameters between Koch and the current study could be compared, including the mean engorgement weight of adult female *A. americanum* (0.42 g), which was lower than that of our study (0.63 ± 0.03). Mean egg-laying success (percent of females that fed to repletion and laid eggs) was higher than observed in our study (84.80% versus 70.50%) and mean larval hatch success (95.30%; percent of egg masses laid that hatch) was similar to that observed in our study (95.08 ± 3.84). Differences could be attributed to the methodologies used, including incubation of engorged females at different temperatures (26°C versus 22°C this study). While both our study and Koch (1988) used tick-naïve captive WTD, geographic differences in host origin were still a factor (Texas versus Oklahoma). Furthermore, our study restricted the tick infestation to the area between the shoulder blades, restricted host grooming, and only infested with the adult life stage, while Koch’s (1988) free-release infested the animals with ticks of all life stages and used twice as many adult pairs. When grooming was restricted, more than 80% of ticks were recovered (this study), whereas < 10% of ticks were recovered from deer allowed to self-groom ticks (Koch 1988). While restricting self-grooming and eliminating allogrooming is artificial, we sought to estimate feeding and reproductive parameters of ticks feeding during artificial infestations on captive WTD with the purpose of serving as a baseline value for comparison to future captive studies and contributing to the body of literature useful for parameterizing mathematical simulation models. Additional experiments should also investigate the degree of self- and allogrooming of ticks by WTD. The impact of concurrent exposure on feeding success is unclear but deserves further study, and our approach could be used to define feeding parameters of larval and nymphal *I. scapularis* and *A. americanum* on WTD.

These data are useful for informing future research regarding interactions between WTD and tick ectoparasites and, coupled with surveys of immature stage and adult tick loads on free-ranging WTD across different geographic regions, could inform/parameterize deterministic or mechanistic models such as LYMESIM 2.0 (Gaff et al. 2020) to determine how this host influences *I. scapularis* and *A. americanum* tick populations in nature.
